# Non-technical skills in pre-hospital care in the Czech Republic: a prospective multicentric observational study (NTS study)

**DOI:** 10.1186/s12873-022-00642-4

**Published:** 2022-05-13

**Authors:** David Peran, Roman Sykora, Jana Vidunova, Ivana Krsova, Jaroslav Pekara, Metodej Renza, Nikola Brizgalova, Patrik Ch. Cmorej

**Affiliations:** 1 Emergency Medical Services of the Karlovy Vary Region, Karlovy Vary, Czech Republic; 2 Prague Emergency Medical Services, Prague, Czech Republic; 3 Department of Anaesthesia and Intensive Care, Third Faculty of Medicine, Charles University and FNKV University Hospital, Prague, Czech Republic; 4 Medical College, Prague, Czech Republic; 5 Emergency Medical Services of the Pilsen Region, Pilsen, Czech Republic; 6 Jan Evangelista Purkyne University, Faculty of Health Studies, Usti nad Labem, Czech Republic; 7 Emergency Medical Services of the Usti nad Labem Region, Usti nad Labem, Czech Republic

**Keywords:** Non-technical skills, Pre-hospital care, Emergency medical services, Communication, Situational awareness, Task management, Decision-making, Teamwork, Team leading

## Abstract

**Background:**

Non-technical skills (NTS) are important for the proper functioning of emergency medical ambulance crews but have hardly been researched in the conditions of clinical pre-hospital care.

The primary objective of this study, therefore, is to describe the use of NTS in practice. The secondary objective is to compare if the performance of NTS varies according to the type of case.

**Methods:**

In this multicentric observational study the modified Team Emergency Assessment Measure (TEAM) score was used to assess the performed NTS of two or more crews on site. The evaluation consisted of leadership, teamwork and task management, rated by a field supervisor.

The study observations took place in real clinical pre-hospital emergency medical cases when two or more crews were dispatched between October 2019 and August 2020. The sample size was determined by researchers prior to the study to at least 100 evaluated events per each of the three participating emergency medical services.

The results are presented as median and interquartile range. The internal reliability, consistency and validity of test items and results were evaluated. The Kruskal–Wallis test and the post hoc Mann-Whitney U test with Bonferroni correction were used for multiple comparisons of three groups.

**Results:**

A total of 359 events were evaluated. Surprisingly, the median value for all eight items was as high as 3.0 with a similar interquartile range of 1.0. There were no differences observed by case type (CPR vs. TRAUMA vs. MEDICAL) except from item 1. A post hoc analysis revealed that this difference is in favour of a higher rated performance of non-technical skills in CPR.

**Conclusions:**

The overall result of the performance of non-technical skills can be regarded as very good and can serve for further evaluations. The crews achieved better parameters of NTS in leadership in resuscitation situations in comparison with general medical events.

**Trial Registration:**

The study is registered at Clinical Trials under the ID: NCT04503369.

## Background

Training of non-technical skills (NTS), including leadership and team training, is used to improve outcomes of cardiopulmonary resuscitation (CPR) [1]. NTS consists of several aspects: (i) teamwork – the work of the team consists of information exchange and coordination of all activities performed, in-team communication is always calm and assertive, and team members support each other; (ii) task management – with the right planning and preparation of individual activities, the likelihood that all standards of care and best practices which will follow increase; (iii) situational awareness – refers primarily to the collection of information, its understanding, and on that basis, prediction of the direction in which the situation will develop; maintaining situational awareness is crucial in order to move towards the right goal; (iv) decision-making – with good situational awareness, one is able to keep in mind all information needed to make the right decision at the right time; in some cases, it is advisable to consult with other team members or to verify their consent to the proposed procedure; (v) communication – a vital tool when working in a team [2]. There is a lack of published studies about the use of NTS in a real medical environment and no articles describing the use of NTS in a real, non-simulated emergency medical services environment [3].

The primary objective of this study is to determine whether Emergency Medical Services (EMS) crews in the Czech Republic are using NTS in practice and, if they do, to describe how. The secondary objective is to compare whether NTS and team performance varies according to the type of case, i.e. resuscitation (CPR), trauma or general medical events.

## Methods

### Design

A prospective, observational multicentric study of NTS in clinical pre-hospital emergencies was conducted. No randomisation was applied.

### Locations

The study took place in three independent organisations in three Czech regions: EMS of Prague, EMS of the Karlovy Vary Region and EMS of the Pilsen Region. The total population of these three regions is approx. 2.2 million [4] with approx. 250,000 emergency cases per year [5]. The data from real clinical events was collected between October 2019 and August 2020.

### Eligible criteria

All EMS cases in the Czech Republic in which pre-hospital care was provided by two or more crews (at least four crew members) and the field supervisor were available for observation. The EMS is organised by the regional government as a rendezvous system – advanced care ambulance with paramedic and emergency medical technician and rapid response vehicle with emergency medical technician (or paramedic) and physician on board. Those units can be supported by a field supervisor in all three regions.

Only those cases where two or more ambulance units/crews met with the presence of the field supervisor were observed and included in the study.

**Fig. 1:**
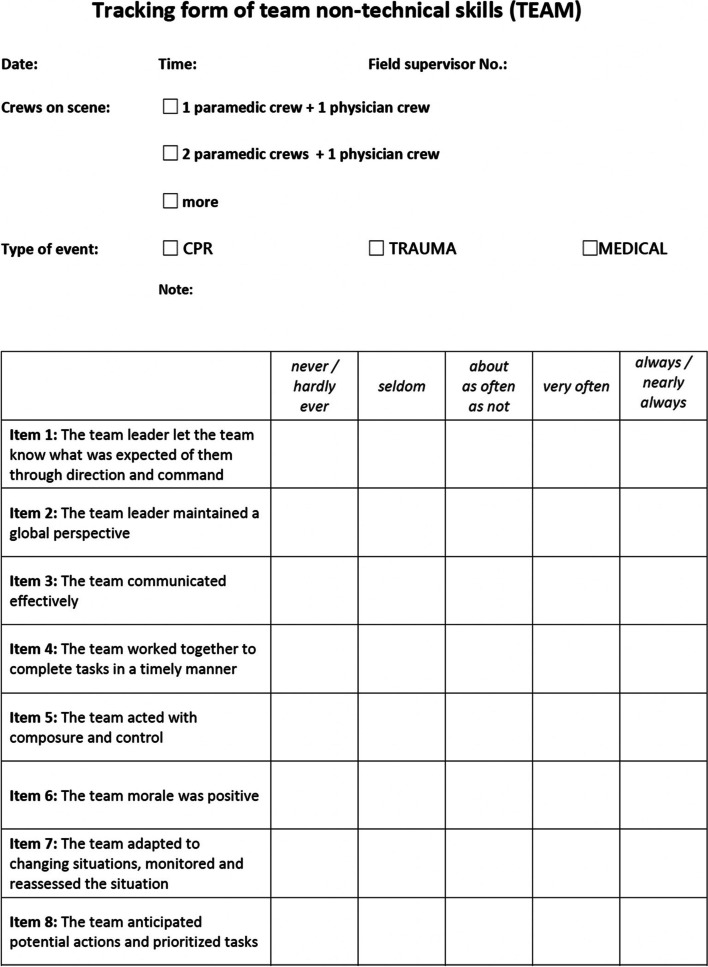
Modified Team Emergency Assessment Measure questionnaire (TEAM). For each item of TEAM questionnaire the rating of presented performance of non-technical skill was noted and subsequently converted to a numeric value and recorded to the final dataset: never/hardly ever = 0; seldom = 1; about as often as not = 2; very often = 3; always/nearly always = 4

In all eligible events, the date and time, the number of crews working in the field (two, three or more), the type of event (CPR, Trauma and Medical, i.e. general medicine emergency events), the name of the field supervisor evaluating the event and the location of the EMS were recorded (Fig. 1).

### Exclusion criteria

All emergency cases with only one crew or events without the presence of the field supervisor were excluded, as well as all cases without complete forms.

### Outcome measures

#### Adaptation of the team emergency assessment measure

The modified and simplified Team Emergency Assessment Measure questionnaire (TEAM) was used in this study (Fig. 1). The creation and validation of this tool is described elsewhere and its validation was not part of this study [6–11]. The TEAM was modified for this study as follows: items one through six, i.e. (1) the team leader let the team know what was expected of them through direction and command, (2) the team leader maintained a global perspective, (3) the team communicated effectively, (4) the team worked together to complete tasks in a timely manner, (5) the team acted with composure and control and (6) team morale was positive, all remained unchanged. Item 7 (the team adapted to changing situations) and item 8 (the team monitored and reassessed the situation) were merged into one item, as were items 9 (the team anticipated potential actions) and 10 (the team prioritised tasks). Item 11 (the team followed approved standards/guidelines) was not used, nor was item 12 (the global score) to simplify the field evaluation in the pre-hospital setting. Moreover this modification has been used in the past to evaluate NTS in simulated scenarios [12], and was therefore well known to researchers and field supervisors.

Each of eight TEAM items were rated using a five-point scale (range 0–4; 0 never / hardly ever, 1 seldom, 2 about as often as not, 3 often, 4 always / nearly always) and covered three categories – leadership, teamwork and task management – the same way as the original TEAM [8]. The total score was calculated as the sum of the values of the eight items and used for further statistical interpretations.

Twenty field supervisors (EMS of Prague, *n =* 5; EMS of the Karlovy Vary Region, *n =* 6; EMS of the Pilsen Region, *n =* 9) underwent a standardised e-learning course on the use of the modified TEAM score before the study began. At the end of the e-learning course, each participating field supervisor had to evaluate video recordings of two simulated clinical scenarios (numbered one and two) with actors for further evaluation of inter-rater variability of field supervisors assessments.

#### Subgroup analysis

This study compared the results of observations between subgroups of cardiopulmonary resuscitation (CPR – defined by the occurrence of cardiac arrest with ongoing CPR on scene), traumatic (TRAUMA – defined by the occurrence of any injury) and general medical events (MEDICAL – defined by any other non-traumatic, non-CPR but general medical situations, including paediatric cases).

### Statistical analysis

Baseline characteristics are reported as numbers and percentages. The results of individual items and the total score of modified TEAM questionnaires are presented as median and interquartile range. Internal reliability, consistency and validity were evaluated through inter-item correlation and Cronbach’s alpha coefficient and item to total correlation [13]. Inter-rater reliability was assessed with intraclass correlation coefficient (ICC) of evaluation of the two different simulated scenarios (video recordings) [14].

The nonparametric Kruskal–Wallis test was used to compare modified TEAM scores among three presented subgroups, with *p* < 0.05 considered as significant. The post hoc Mann-Whitney *U* test with Bonferroni correction was used for multiple comparisons.

The sample size was not calculated but was determined by researchers prior to the study to at least 100 evaluated events per each participating emergency medical services.

The data were collected and basic calculations performed in Excel (Microsoft, USA). Statistical software *STATISTICA* 7.0 (StatSoft, USA) was used for statistical analyses and calculation. The ICC calculation software Mangold, Pascal (2018), based on Wirtz & Caspar 2002, (Germany) was used to calculate the adjusted average scores, assuming no interaction effect was present.

### Reliability, consistency and validity of testing of modified TEAM score

The inter-rater variability assessed by ICC was 0.958 for e-learning scenario number 1 and 0.701 for e-learning scenario number 2. Inter-item correlation for items 1–8 varied from 0.53 to 0.78, with average inter-item correlation 0.63. Cronbach’s alpha coefficient of the final dataset was 0.93 and item-total correlations varied from 0.79–0.87.

## Results

**Fig. 2:**
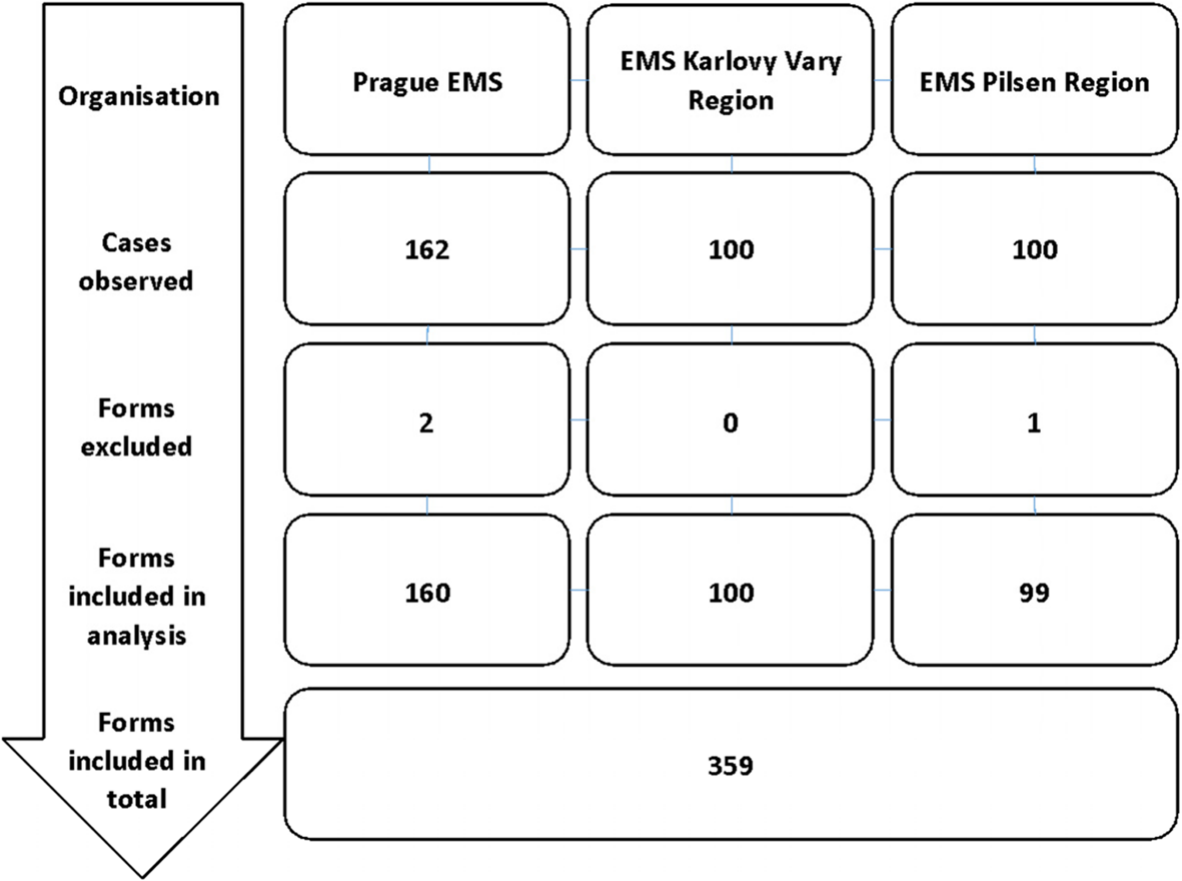
Study flow diagram

A total of 359 events were evaluated in the study after three events were excluded due to incomplete data (Fig. 2). The case types were represented differently between the three different EMS: medical events were the most common category in the EMS of the Karlovy Vary Region, but hardly seen in the Prague EMS, and in the Pilsen Region the categories were more evenly distributed. However, these differences were no longer evident in the total number of events monitored (CPR, *n =* 110 vs. TRAUMA, *n =* 122 vs. MEDICAL, *n =* 127). The most frequently evaluated events were when two ambulances intervened, i.e. with four crew members on the spot (*n =* 317; 88%) (Table 1).

**Table 1:** Characteristics of events

	Prague EMS (*n =* 160)	EMS of Karlovy Vary Region (*n =* 100)	EMS of Pilsen Region (*n =* 99)	TOTAL (*n =* 359)
Event type:				
CPR	72 (45%)	11 (11%)	27 (27%)	110 (31%)
Trauma	84 (53%)	8 (8%)	30 (30%)	122 (34%)
Medical	4 (2%)	81 (81%)	42 (43%)	127 (35%)
Number of crews:				
Two	142 (88%)	99 (99%)	76 (77%)	317 (88%)
Three	12 (8%)	1 (1%)	16 (16%)	29 (8%)
More	6 (4%)	0	7 (7%)	13 (4%)

Data are presented as number of cases and percentage (if appropriate); *CPR C*ases of cardiac arrest with ongoing cardiopulmonary resuscitation, *n =* number of cases

One ambulance crew means two members of the crew either with a physician and an emergency medical technician/paramedic (the physician crew) or two members of the crew without a physician (the paramedic crew)

### Results of the overall “modified TEAM SCORE”

Monitoring using a modified TEAM score for the whole set of events showed their high use of non-technical skills. Surprisingly, the median value for all eight items was 3.0 with a similar interquartile range. No major shortcomings or reductions in the values of any of the skills expressed in the individual items were observed. The overall modified TEAM ratings including total score are presented in detail in Table 2.

**Table 2:** Overall modified TEAM rating outcomes

	Item statistics	
	Median (IQR)	Observed range
**Item 1:** The team leader let the team know what was expected of them through direction and command	3.0 (2.0–3.0)	0–4
**Item 2:** The team leader maintained a global perspective	3.0 (2.0–3.0)	0–4
**Item 3:** The team communicated effectively	3.0 (2.0–3.0)	0–4
**Item 4:** The team worked together to complete tasks in a timely manner	3.0 (2.0–3.0)	0–4
**Item 5:** The team acted with composure and control	3.0 (2.0–3.0)	0–4
**Item 6:** Team morale was positive	3.0 (2.0–4.0)	0–4
**Item 7:** The team adapted to changing situations, monitored and reassessed the situation	3.0 (2.0–3.0)	0–4
**Item 8:** The team anticipated potential actions and prioritised tasks	3.0 (2.0–3.0)	0–4
**Total score**	23.0 (19–27)	

Data are presented as median and interquartile range (IQR; 25th percentile to 75th percentile); *p* values are presented for item-test (item-total) correlations. TEAM: Team Emergency Assessment Measure with subsequent ratings of presented performance of non-technical skill: 0 = never / hardly ever; 1 = seldom; 2 = about as often as not; 3 = very often; 4 = always / nearly always

### Results of the “modified TEAM score” by type of case

The presented data were very similar when comparing the modified TEAM score by case type (CPR vs. TRAUMA vs. MEDICAL). The Only significant difference was observed in item 1 (The team leader let the team know what was expected of them through direction and command) H (2; 359) = 7.64, *p* = 0.02. A post hoc analysis revealed that this difference is in favour of a higher rated performance of non-technical skills in CPR than in MEDICAL events: U (NCPR = 110, NMEDICAL = 127) = 5627, Z = − 257,990, *p* = 0.0099, and none of the other pairwise comparisons were significant after Bonferroni adjustment (all *p* > 0.17). Similarly, despite tendency to better results in item 4 (The team worked together to complete tasks in a timely manner; *p* = 0.06) and item 5 (The team acted with composure and control; *p* = 0.09) this result did not reach statistical significance and therefore no post hoc multiple comparisons analysis was performed. There were no differences observed among other items and total score (Table 3).

**Table 3:** Comparison of modified TEAM score by case type

	CPR (*n =* 110)	TRAUMA (*n =* 122)	MEDICAL (*n =* 127)	
**Item 1:** The team leader let the team know what was expected of them through direction and command	3.0 (2.0–4.0)*	3.0 (2.0–3.0)	3.0 (2.0–3.0)*	*p =* 0.02
**Item 2:** The team leader maintained a global perspective	3.0 (2.0–3.0)	3.0 (2.0–3.8)	3.0 (2.0–3.0)	p = 0.32
**Item 3:** The team communicated effectively	3.0 (2.0–3.0)	3.0 (2.0–3.0)	3.0 (2.0–3.0)	p = 0.70
**Item 4:** The team worked together to complete tasks in a timely manner	3.0 (3.0–3.0)	3.0 (2.0–3.0)	3.0 (2.5–3.0)	*p =* 0.06
**Item 5:** The team acted with composure and control	3.0 (2.0–4.0)	3.0 (2.0–3.0)	3.0 (3.0–4.0)	*p =* 0.09
**Item 6:** Team morale was positive	3.0 (2.0–4.0)	3.0 (2.0–4.0)	3.0 (2.0–3.0)	p = 0.62
**Item 7:** The team adapted to changing situations, monitored and reassessed the situation	3.0 (2.0–4.0)	3.0 (2.0–3.0)	3.0 (3.0–3.0)	*p =* 0.33
**Item 8:** The team anticipated potential actions and prioritised tasks	3.0 (2.0–3.0)	3.0 (2.0–3.0)	3.0 (3.0–3.0)	*p =* 0.10
**Total score**	24.0 (19.3–28.0)	22.5 (17.3–27.0)	23.0 (20.0–26.0)	*p* = 0.37

Data are presented as median and interquartile range (25th percentile to 75th percentile); *p* values are presented for Kruskal Wallis test; * indicates the significant difference between marked subgroups in pairwise comparisons by Mann Whitney U test with Bonferroni correction; CPR Cases of cardiac arrest with ongoing cardiopulmonary resuscitation, *n* = Number of cases

## Discussion

The results showed high use of the NTS on an average level and there was no difference observed among the subgroups of different medical conditions.

So far, similar results were published only from the hospital environment or simulations or clinical situations with a focus on one part of the non-technical skills or during events of out-of-hospital cardiac arrest only [6, 15–17]. The presented data are comparable in terms of consistency and reliability, but also in terms of the values of the results with the original work, and sufficient data consistency and interrater variability was observed, calculated and published [7–11, 16, 18]. The research team used the complete TEAM form for pilot field observations [12]. However, based on the pilot findings, the researchers used a simplified form for field supervisors to work in pre-hospital care. In this study we evaluated the situation on the spot, not later, for example from a video recording [17].

This baseline characteristic of NTS obtained from three independent emergency medical services organisations from different regions in the Czech Republic (from the urban region of the capital, rural and mountainous areas, and from a region where urban and rural characteristics of pre-hospital care are combined) provides awareness of the starting level of staff who have not yet been trained in non-technical skills. The specific reason for the study was not to monitor the clinical performance of the provided care, but to focus on NTS. We did not focus on the correlation between non-technical skills performance and clinical outcome.

From the outset, there was no intention to compare individual EMS but to create a suitable case mix, which could correspond with the global spectrum in pre-hospital emergency care in the Czech Republic. The best results were observed especially in CPR, mainly due to significantly better leadership. CPR is “strictly” algorithm-driven, and thus it might be easier for the team leader to instruct the crew members and for team members to follow. Our results that are completely different from those of a recently published study of 114 cases of hospital cardiac arrest, where the leadership was evaluated as the worst [17]. This situation can be partly explained by the fact that the most common training, where aspects of NTS and leadership are also explained, are the Advanced Life Support courses of the European Resuscitation Council, which are widespread in the Czech Republic. This result may also indicate that the focus on the standardised approach is also needed in other fields like trauma and general medical care. Except for CPR, other events observed in the study had very different clinical characteristics and the main goal was to evaluate the aspects of NTS in the interplay of multiple field teams rather than the overall competence. However, the authors are aware that the evidence indicates that checklists tend to overlook the more holistic components of clinical competence, suggesting that global ratings of performance are appropriate [11, 19, 20]. In this context, there is the question of how the scale is set in the validated TEAM score and whether differences in team performance or algorithm application are being revealed. So far, most of the works for individual items report an average score or median around 3 and it is very rarely published whether the level of NTS measured by TEAM score correlates with clinical outcome. In this study, the non-technical skills scale for trauma (T-NOTECHS) [21, 22] or other teamwork assessment measures was not used despite NTS being evaluated in the subgroup of trauma patients. This was mainly to have only one tool for evaluation and the TEAM tool is also highly effective for assessing trauma [11].

### Limitations

When adapting the TEAM forms for this study, the researchers merged some TEAM items and omitted the global score as set by Cooper et al. mainly to simplify the evaluation and focus purely on individual aspects of NTS in real clinical field work in a clinically dynamic environment.

As TEAM can be regarded as a proven method, only a data consistency check was performed prior to statistical evaluation. Interrater variability was determined by evaluating the observer’s video scenarios and not to a certain proportion of actual field cases, where 10% of cases were considered normal and was done in the original validation study [8]. This was not feasible in a multicentric field study due to logistical reasons. The relatively high number of field supervisors (*n* = 20) could be regarded as a limitation, but their training in TEAM evaluation was extensive and resulted in low interrater variability, which had been checked and proved [11, 23].

A major limitation of this study is that no sample calculation was performed prior to the start of the study. It was only decided to obtain 100 measurements from each participating EMS. This shortcoming in the performed power analysis coupled with the above-mentioned ambiguities in the stratification of the TEAM score scale may cause some uncertainty in the presentation of the results. For now, it is necessary to take these results as a pilot and to follow up the research of other issues such as the correlation of NTS and clinical outcomes, learning effects or standardisation of algorithms.

## Conclusions

The overall result of non-technical skills performance can be regarded as very good and can serve for further evaluations. There was no significant difference observed according to the medical conditions of the events except for better leadership in CPR.

## Data Availability

All data generated or analysed during this study are included in this published article. The baseline dataset used and analysed during the current study is available from the corresponding author on reasonable request.

## Abbreviations

CPR: Cardiopulmonary resuscitation
NTS: Non-technical skills
TEAM: Team Emergency Assessment Measure questionnaire
EMS: Emergency Medical Services
ICC: Intraclass correlation coefficient
